# Bilateral lesion of the cerebellar fastigial nucleus: Effects on smooth pursuit acceleration and non-reflexive visually-guided saccades

**DOI:** 10.3389/fneur.2022.883213

**Published:** 2022-09-20

**Authors:** Christoph Helmchen, Björn Machner, Hannes Schwenke, Andreas Sprenger

**Affiliations:** ^1^Department of Neurology, University Hospitals Schleswig-Holstein, Campus Lübeck, Lübeck, Germany; ^2^Center of Brain, Behavior and Metabolism (CBBM), University of Lübeck, Lübeck, Germany; ^3^Department of Neuroradiology, University Hospitals Schleswig-Holstein, Lübeck, Germany; ^4^Institute of Psychology II, University of Lübeck, Lübeck, Germany

**Keywords:** initial smooth pursuit, fastigial nucleus, cerebellum, saccade hypermetria, bilateral

## Abstract

**Background:**

“Central dizziness” due to acute bilateral midline cerebellar disease sparing the posterior vermis has specific oculomotor signs. The oculomotor region of the cerebellar fastigial nucleus (FOR) crucially controls the accuracy of horizontal visually-guided saccades and smooth pursuit eye movements. Bilateral FOR lesions elicit bilateral saccade hypermetria with preserved pursuit. It is unknown whether the initial acceleration of smooth pursuit is impaired in patients with bilateral FOR lesions.

**Objective:**

We studied the effect of a cerebellar lesion affecting the deep cerebellar nuclei on the initial horizontal pursuit acceleration and investigated whether saccade dysmetria also affects other types of volitional saccades, i.e., memory-guided saccades and anti-saccades, which are not performed in immediate response to the visual target.

**Methods:**

We recorded eye movements during a sinusoidal and step-ramp target motion paradigm as well as visually-guided saccades, memory-guided saccades, and anti-saccades in one patient with a circumscribed cerebellar hemorrhage and 18 healthy control subjects using a video-based eye tracker.

**Results:**

The lesion comprised the FOR bilaterally but spared the posterior vermis. The initial pursuit acceleration was low but not significantly different from the healthy control subjects and sinusoidal pursuit was normal. Bilateral saccade hypermetria was not only seen with visually-guided saccades but also with anti-saccades and memory-guided saccades. The final eye position remained accurate.

**Conclusion:**

We provide new insights into the contribution of the bilateral deep cerebellar nuclei on the initial acceleration of human smooth pursuit in midline cerebellar lesions. In line with experimental bilateral FOR lesion data in non-human primates, the initial pursuit acceleration in our patient was not significantly reduced, in contrast to the effects of unilateral experimental FOR lesions. Working memory and neural representation of target locations seem to remain unimpaired. Our data argue against an impaired common command feeding the circuits controlling saccadic and pursuit eye movements and support the hypothesis of independent influences on the neural processes generating both types of eye movements in the deep cerebellar nuclei.

## Introduction

“Central dizziness” can be caused by cerebellar disease. In the absence of vestibular abnormalities (e.g., spontaneous nystagmus, gaze-evoked nystagmus) or limb ataxia, oculomotor abnormalities can be the only signs indicative of a cerebellar disease in a very specific way. Here, we present a rare case history of a bilateral fastigial nucleus lesion that reveals new insights into the functional role of the patient's initial pursuit generation, i.e., pursuit acceleration. To our knowledge, this has not been examined in previous patient studies.

### Role of the cerebellum on saccades and smooth pursuit eye movements

As the cerebellar neural control of both smooth pursuit eye movements and saccades rest upon bilateral fastigial nuclei activity (1–3), unilateral lesions of the oculomotor parts of the cerebellar vermis (OMV) and the underlying deep cerebellar nuclei, specifically the fastigial nuclei (FOR), elicit unilaterally impaired smooth pursuit and direction-specific saccade dysmetria, which can be clinically recognized. Even inexperienced clinicians recognize saccade dysmetria as the saccade hypometria in one direction is contrasted by hypermetria on the other side.

In bilateral cerebellar lesions, however, clinical signs become more difficult to be identified as the balance of the abnormal driving forces counterbalance each other and may even elicit normal appearing signs (normal smooth pursuit) (4), bilateral saccade hypermetria in bilateral fastigial lesions (5) or bilateral hypometria in OMV lesions (6). On clinical examination, saccade dysmetria, in this case, is usually missed because the pathological dysmetria, in particular in saccade hypermetria, does not appear different from the dysmetric saccade toward the contralateral side, pretending a normal saccade behavior. While the latter effects have been elicited by posterior vermis (7) and FOR (4, 5) inactivation studies in non-human primates, they have been replicated in single patient studies with vermal and FOR lesions (8–11). The opposite effects of lesions in both structures, the oculomotor vermis (OMV) and the FOR, are possibly related to the inhibitory control of the Purkinje cells of the OMV on the FOR (12).

### Role of the fastigial nucleus on smooth pursuit eye movements

The activity of smooth pursuit neurons in the FOR is direction-specific and encodes eye acceleration: as their discharge precedes the time of peak eye velocity during contralateral movements, these neurons have been functionally linked to eye acceleration. Neurons with preferred modulation of their discharge rates during ipsilateral smooth pursuit lag peak velocity and discharge during eye deceleration (13). Assuming a role of the FOR in accelerating contralateral and decelerating ipsilateral smooth pursuit, it remained unclear in the few related studies whether eye acceleration during the initial period of pursuit (i.e., after a target has started moving or has changed its speed) is impaired in patients with FOR lesions, as bilateral FOR lesions would suppress the imbalance between the sustained activity emitted by both FOR.

The pursuit acceleration in the initial, open-loop phase of pursuit tracking behavior seems to be selectively vulnerable in some patients with cerebellar lesions (14) but the lesion site remained unraveled and has not been examined in patients with circumscribed FOR lesions yet (8–10). In non-human primates, bilateral experimental FOR lesions do not or only mildly reduce the initial acceleration of smooth pursuit (4). However, an impairment has been shown in lesions of the oculomotor vermis, possibly related to asymmetrical lesions (7).

Noticeably, as pursuit and saccade fibers cross the midline at the rostral level and project to the contralateral side, unilateral FOR lesions may have bidirectional effects. A recent anatomical study has shown inter-fastigial projections along the roof of the fourth ventricle in mice (15) but these projections have neither been identified in the non-human primate yet nor been functionally characterized as related to the control of eye movements.

Based on these animal studies, we tested the hypothesis that unpredictive initial acceleration of smooth pursuit is not impaired in a patient with a circumscribed bilateral lesion of the deep cerebellar nuclei, specifically involving the FOR. For better comparison with previous studies, we also tested predictive sinusoidal pursuit behavior. Importantly, this patient's lesion spared the oculomotor vermis and the flocculus. The patient's eye movements were compared with 18 healthy control subjects to investigate the role of this patient's FOR in the initial pursuit acceleration.

### Role of the fastigial nucleus on saccadic eye movements

Both structures, the OMV (6, 16) and the FOR (17), crucially control the accuracy of visually-guided saccades (18). Purkinje cells of the OMV (lobules VI and VII) contain saccade-related neurons (19, 20) and lesions elicit uni- (6) or bilateral saccade hypometria and increased trial-to-trial variability of saccade amplitude (21). Unilateral FOR lesions in animals cause direction-specific saccade deficits: contralesional hypometria and ipsilesional hypermetria in the head restrained (2, 5, 22) and unrestrained (23, 24) conditions. Dysmetria affected the horizontal components of saccades in all directions. These direction-specific oculomotor signs can be clinically recognized in patients with direct or indirect FOR lesions (25). In contrast, bilateral FOR lesions elicit severe bilateral saccade hypermetria during visually-guided saccades, in non-human primates (5) and patients (9, 11, 26).

Dysmetria is not only seen during visually-guided saccades to stationary but also to moving targets and interceptive saccades follow the same directional dependence (2, 3), with hypometric contralesional and hypermetric ipsilesional saccades. Moreover, saccade-related burst neurons in the FOR are not only active during visually-guided and memory-guided saccades but also during spontaneous saccades in light and darkness (27), although not always (28). Volitional saccades may be initiated during visual scanning as part of a visual recognition or natural orientation behavior to remembered or estimated target locations (29). Saccade dysmetria was not found during internally triggered saccades of a patient scanning a set of targets (9). In order to look at targets of interest that one recognizes during visual scanning, the subject must encode and remember the location of the (peripheral) target. Like in other visually-guided saccades, potential changes in eye position must be taken into account for accurate orientation of gaze before the saccade to the remembered target location is executed (10). This function engages control functions of disengaging from the fixated target, maintaining gaze direction during fixation, suppressing looking at distracting targets, and looking as precisely as possible at the remembered location. These functions crucially involve the frontal cortex with the supplementary (SEF) and frontal eye field (FEF), dorsolateral prefrontal cortex (DLPFC)(30, 31), and the cerebellum, particularly the OMV and the underlying deep cerebellar nuclei, specifically the FOR (32, 33). Up to now, only a few studies investigated memory-guided saccades in cerebellar disease (10, 26, 34). Memory-guided saccades were found to be as dysmetric as visually-guided saccades (10, 26) or even more dysmetric (34). However, these studies did not show lesions constrained to the deep cerebellar nuclei. A recent study examined saccade hypermetria of a patient with bilateral FOR lesion sparing the vermis but did not examine saccades toward remembered visual targets (memory saccades) or anti-saccades (11).

Anti-saccades are directed to the opposite side of the presented stationary target (35, 36) which are usually examined in patients with frontal lobe and basal ganglia disease (37, 38) or schizophrenic patients (39) but not in cerebellar disease.

In memory-guided saccades, an efference copy signal is usually not needed toward a memorized visual target since no motor command is generated when the target is presented. It is a matter of debate whether non-visual, extra-retinal signals (e.g., efference copy) could influence the programming of the direction and accuracy of memory-guided saccades (10). Cerebellar patients have been suggested to lack an efference copy of the eye position after the first saccade due to the lack of corrective saccades in the dark (34). In line with this notion, dysmetria of memory-guided saccades increase once the eyes move during the memory period (10). The authors suggested that an efference copy could come into play not as a precise record of the motor command but as a cue to re-evaluate the visual consequences of the saccade.

Both types of saccades are endogenously driven voluntary saccades and engage different mechanisms and neural networks (29). Subjects have to look at an imagined target position during the anti-saccade paradigm without having seen a visual target at this location. In a memory-guided paradigm, they have to keep the target position in mind within a variable interval, challenging working memory. Thus, the execution follows a mental representation of a target that is no longer visible. If non-visual signals influence saccade execution under these circumstances, the magnitude of dysmetria may differ between on the one hand pro-saccades, on the other hand, memory-guided and anti-saccades.

We hypothesized that saccade dysmetria in our patient with a bilateral FOR lesion differs between memory-guided, anti-saccades, and visually-guided saccades.

## Methods and participants

### Participants

Oculomotor data of a 43-year-old man were compared with 18 healthy subjects (age: 38 ± 8 years, mean ± standard deviation). The study was approved by the Ethics Committee of the University of Lübeck (AZ12-219), and all participants gave written informed consent.

#### History of present illness

The formerly healthy patient complained about sudden headache, dizziness, blurred vision, and pronounced unsteadiness of stance and gait with some short-lasting slurring of speech. He did not notice oscillopsia or lateropulsion. On examination, there was severe saccade hypermetria bilaterally to foveopetal and foveofugal visual targets. Horizontal and vertical smooth pursuit appeared normal. Slow di- and convergence was slightly cogwheel. Transient horizontal nystagmus was reported in the emergency room that could not be identified any longer a few hours later. Horizontal and vertical head-impulse testing was normal. There was no head-shaking, gaze-evoked, positional, or rebound nystagmus. Past medical history was otherwise unremarkable. There was postural unsteadiness with a slightly broad-based stance in the light, which increased on eye closure, but there was no ataxia of the extremities.

Clinical MRI (Siemens Vida 3 T MRI, Erlangen, Germany) was performed with a hospital-specific cerebral hemorrhage detection protocol. On high-resolution FLAIR images (voxel size 1 mm, TR 6,500 ms, and TE 393 ms), the deep cerebellar nuclei were localized by means of triplanar reconstruction, with the strict matching of the hypointense deep cerebellar nuclei regions by means of clinical atlases (40). Furthermore, in the same manner, anatomical localization of the cerebellar lobules was performed (41).

### Experimental setup and oculomotor paradigms

Eye movements were recorded with a video-based eye-tracker (Eyelink II, SR Research Ltd., Ontario, Canada). We recorded both eyes but only movements of the left eye were analyzed. The fixation target was placed straight ahead of the nose. During experiments, subjects sat in a comfortable chair; the head was immobilized by a chin rest and a forehead-holding device. The visual stimulus consisted of a red laser dot (diameter of 0.1°), rear-projected onto a translucent screen at a viewing distance of 1.4 m. The laser dot was moved by two galvanometer scanners (GSI Lumonics, Munich, Germany), driven by an analog output card in the stimulus PC (AT-AO6/10, National Instruments). Except when otherwise mentioned, subjects were asked to look at the laser dot as fast and accurately as possible. For calibration, we first presented a sequence of stimuli in central, horizontal, and vertical deflected positions. Recordings were performed in darkness. Visual acuity was >0.8, including the patient (inclusion criteria).

We investigated all participants (patient and healthy subjects) under head-stationary conditions in the dark using the following paradigms which are described in detail elsewhere (37): fixation at gaze straight ahead and on vertical and horizontal eccentric gaze positions (10°, 20°), reflexive horizontal and vertical visually guided saccades (10°, 15°) to a small laser target (VGS = pro-saccades), volitional saccades, i.e., anti-saccades (10°, 15°), and saccades to memorized (“imagined”) target locations (memory-guided saccades; 10°, 15°), as well as smooth pursuit paradigms.

The data analysis was performed in MATLAB^®^ (R2021b, The Mathworks, Natick/MA).

Sinusoidal smooth pursuit paradigms The predictive, closed-loop smooth pursuit was tested in a sinusoidal smooth pursuit paradigm composed of horizontal oscillations of 0.2 Hz (amplitudes of 15.9°; i.e., maximum stimulus velocity of 20°/s; 4 cycles). After the elimination of saccades, the phase and amplitude of a sinusoid were adjusted to match the slow phase velocity of the eye. The fitting was performed with the least-squares method. The gain was calculated by the ratio of eye velocity to target velocity.

The step-ramp paradigm (42, 43) was used as described before (37, 44). This paradigm was used to quantify the initial response of unpredictive smooth pursuit without visual feedback (open loop) and the closed-loop period. During each trial, the target stepped away horizontally from the gaze straight ahead position and then moved with a constant velocity in the opposite direction. Because we used step amplitudes of 2.4° and ramp velocities of 16°/s, the stimulus passed the center after 150 ms, thus allowing smooth pursuit initiation without an initial saccade. Each sequence consisted of 20 ramps to either side in random order. Foveofugal ramps (4 to the left and 4 to the right) with horizontal target steps away from the center position and consecutive constant velocity stimuli in the same direction were interspersed to keep the level of attention high. The duration of the fixation interval before each trial was varied from 1,600 to 1,900 ms randomly (44).

#### Analysis of pursuit acceleration

The onset of pursuit acceleration (pursuit latency) was defined as the time when eye velocity exceeded 3.2 times the standard deviation of the baseline velocity signal (measured over a 200-ms interval before the target started to move). Subsequent data in a 60 ms time window were used to calculate the slope of a least square fit (robust fit function within Matlab^®^) of eye velocity (43), as described in detail by one of us (AS) before (45). The intersection (green dot in **Figure 3**) between the regression line (orange line) and mean eye velocity before target motion onset (blue line) indicates the start of the pursuit eye movement. The slope (of the orange line) indicates pursuit acceleration.

##### Pro-saccades

Gain, latency, and velocity of pro-saccades to visual targets at different locations and displacements (10°, 15°) were examined for the horizontal and vertical directions (30 saccades per direction). Direction and amplitude were randomized, the preceding fixation phase was varied from 1,000 to 1,400 ms followed by a gap of 200 ms. Saccade amplitude gain was calculated as the ratio between the amplitudes of the primary saccade and the target displacement.

As the subjects had to fixate the target for some 1,000–1,400 ms (and a gap of 200 ms) before the next target displacement, eye position was usually on target. However, target retinal eccentricity became important for correction saccades, as the correction saccades of the patient started from an eye position several degrees off the target position (offset). We calculated the amplitude gain of the secondary saccade, i.e., the first correction saccade toward the visual target position (e.g., 10° right or left) as the ratio of the eye amplitude to the distance between the eye position at the start of the secondary saccade and the actual target position. The patient's hypermetria of the correction saccades is better reflected by the mean amplitude of the correction saccades than by the mean amplitude gain. We, therefore, report both values. Furthermore, we prefer to use the term “correction saccade” instead of “corrective saccade” since the patient's saccades following the primary saccade do not always correct the position error.

Latency was the interval from stimulus to saccade onset. Eye velocity was calculated by [difference of median eye position of five data points before and after the actual data point] ^*^ sampling rate (1,000 Hz).

The main sequence of all visually guided saccades was fitted as in previous studies (44, 46) by the common equation: Peak velocity = vmax ^*^ (1- ^e−amplitude/c^) using the *fminsearch* function within Matlab^®^. Using the parameters derived from the fit, we calculated saccade peak velocity for a 15° saccade amplitude. The rationale for this procedure was to compare the peak velocities between the patient and the healthy controls who differed in saccade amplitude: the peak velocity of the patients' saccades was transformed into those expected for saccades with the amplitudes of the healthy controls, i.e., we used transformed eye velocities.

##### Anti-saccade paradigm

While fixating the gaze-straight ahead target, participants were asked not to look as it jumps sideways at 10° and 15° horizontally to the right or left side (direction at random order) but to look in the opposite direction with the same amplitude after a gap (200 ms). We presented 20 steps in each direction. The percentage of errors (error rate, i.e., misdirected saccades toward the target) and latencies of correct saccades were calculated. Anticipatory saccades (*latency*<*70 ms)* were excluded (44).

##### Memory-guided saccade paradigm

In the memory-guided saccade paradigm (30 trials), subjects not only had to suppress reflexive saccades toward the presented lateral target but also had to keep the location in mind for the consecutive task. While they fixated the laser in its initial position at gaze straight ahead, an additional target was flashed for 200 ms at a 10° or 15° peripheral position left or right from the center of the visual display (random direction and unpredictable). The subjects were instructed not to look at the peripheral target but to keep its position in mind within a variable interval (1,500, 2,500, and 3,500 ms). When the central fixation point was switched off, they had to look at the remembered, previously shown target position. After an additional 2 s, the peripheral target showed up again at the previously flashed location and subjects had to fixate this visible target. The number and amplitude of correction saccades toward the remembered target position and the difference between the final eye position and the correct position of the memorized target were analyzed. Fifteen trials in each direction were performed. The percentage of reflexive misdirected saccades toward the flashed target (error rate), the latency, and the amplitude of the first memory-guided saccade were analyzed. To evaluate the accuracy of spatial memory, we calculated the final eye position error, defined as the distance between the eye position at target reappearance and the target position. Accordingly, the final eye position gain is the final eye position error divided by the target amplitude.

### Statistical analysis

Horizontal and vertical eye positions were analyzed using Matlab^®^ (R2021b, The Mathworks Inc., Natick, MA, USA). We used statistical comparisons by the Revised Standardized Difference Test developed for suspected impairments and dissociations in single-case studies (47). If not stated otherwise, subsequently reported values are means (±1 standard deviation).

## Results

### Lesion pattern

**Figure 1:**
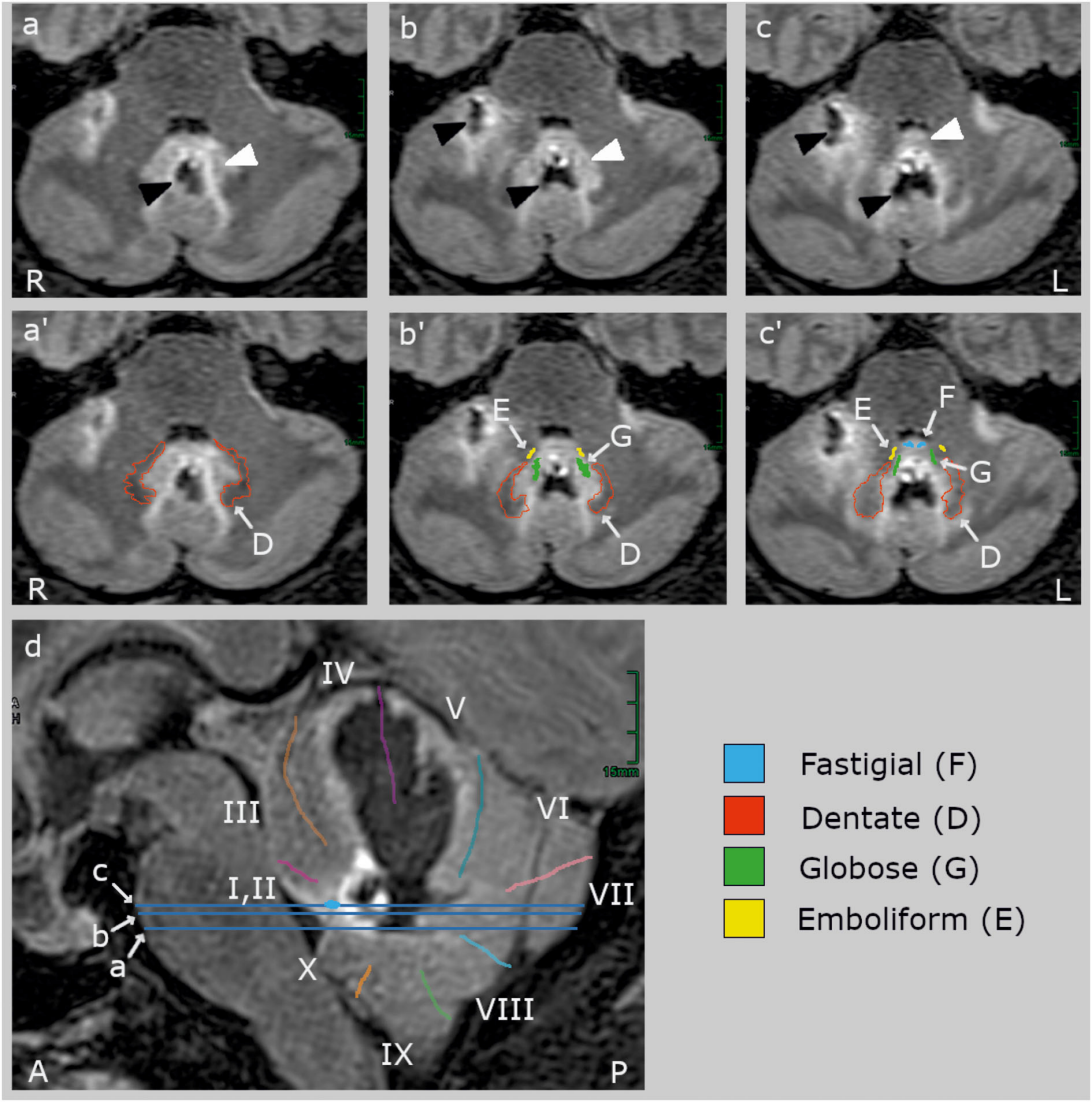
Clinical high-resolution FLAIR images (Siemens Vida 3 T MRI, Erlangen, Germany) with axial **(a–c)** and sagittal slices **(d)**. The blue lines on the sagittal slice indicate the location of the axial slices. The upper row **(a–c)** shows the lesion (black arrowheads) and its edema (white arrowheads) without cerebellar landmarks which are labeled in the middle **(a'–c')** and lower row **(d)**. The hemorrhage (hypointense, black) centered in the midline between the deep cerebellar nuclei and extended rostrally into the vermal lobules IV and V and the right hemispheric lobules V and VI (40, 41). The edema (hyperintense, light gray) involved bilaterally the fastigial nucleus (F, blue) anteriorly to the hemorrhage at the roof of the fourth ventricle, most likely impairing inter-fastigial projections (15), and laterally the interpositus composed of the globose (G) and emboliform (E) nuclei, and the medial part of the dentate nucleus (D). The lesion did not comprise (oculomotor) hemispheric lobule VI (simple lobule), the posterior oculomotor vermal lobules VI and VI (OMV), flocculus, paraflocculus, and caudal vermal uvula and nodulus. There was no brainstem lesion.

MRI identified a hemorrhage with surrounding edema at the level of the deep cerebellar nuclei, involving bilateral fastigial nuclei, extending laterally to the globose and emboliform nuclei on both sides and the medial aspects of the dentate nuclei (Figure 1). The bleeding extended into the anterior vermis (lobules IV and V) but spared the oculomotor vermis (lobules VI, VII) (40, 41). There was no brainstem lesion.

A summary of the important eye movement data is listed in Table 1.

**Table 1:** Horizontal eye movementparameters of the patient and the healthy subjects, with the mean (patient) and the mean (±standard deviation) of the median of the healthy subjects, the number of measurements (N), and the level of statistically significant differences.

**Oculomotor paradigms**	**Patient**	***N***	**Healthy subjects** **(*n* = 18)**	***N***	**Level of** **significance**
**Initial smooth pursuit (step ramp)**		40		40	
Latency (ms)	192		245 ± 62		n.s.
Initial acceleration (°/s^2^)	36		91 ± 43		n.s.
Catch-up saccade (°) during pursuit	3.29 ± 2.1		1.56 ± 1.2		n.s.
**Pro-saccades**		30		30	
Gain	1.59 ± 0.08		0.96 ± 0.06		*p* = 0.001
Gain (correction saccade)	1.1 ± 0.3		0.89 ± 1.22		n.s.
Amplitude (°) of correction saccade	−6.4 ± 7.0		2.4 ± 1.5		*p* = 0.001
Velocity (°/s)	398 ± 57		377 ± 48		n.s.
Latency (ms)	214 ± 68		182 ± 33		n.s.
**Anti-saccades**		40		40	
Error rate (%)	20		24.07		n.s.
Gain	1.47 ± 0.45		0.83 ± 0.22		*p* = 0.007
Gain (correction saccade)	0.64 ± 0.36		0.48 ± 0.21		n.s.
Amplitude (°) of correction saccade	−2.92 ± 5.8		0.84 ± 1.68		*p* = 0.023
Latency	400 ± 73		328 ± 53 ms		n.s.
**Memory-guided saccades**		30		30	
Reflexive saccade (%)	16.6		16 ± 11.5		n.s.
Gain	1.14 ± 0.34		0.84 ± 0.68		*p* = 0.001
Gain (correction saccade)	0.71 ± 0.44		0.65 ± 0.26		n.s.
Amplitude (°) of correction saccade	−2.3 ± 1.9		0.8 ± 0.9		n.s. (*p* = 0.06)
Final eye position error (°)	0.98 ± 0.26		0.93 ± 0.05		n.s.
Variability (final eye position)	0.26		0.19 ± 0.11		n.s.
Latency (ms)	322 ± 100		452 ± 110		n.s.

### Smooth pursuit

#### Pursuit maintenance

The sustained smooth pursuit was analyzed both in the step-ramp and in the sinusoidal pursuit paradigm.

**Figure 2:**
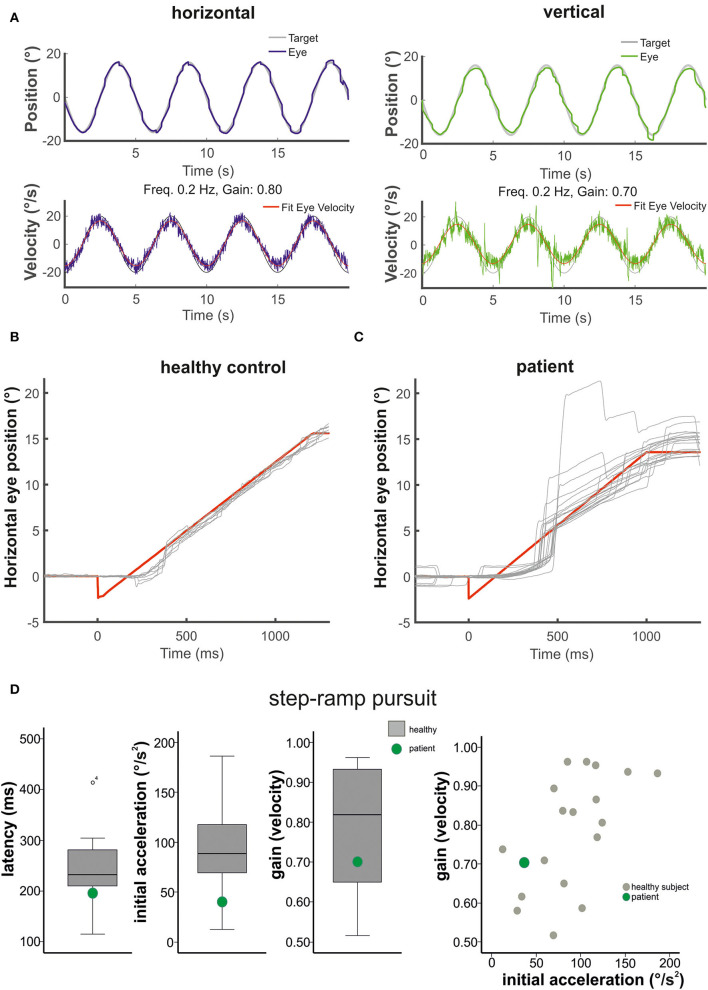
The figure shows in **(A)** the horizontal (left) and vertical (right) sinusoidal smooth pursuit of the patient. Upper traces indicate horizontal (blue) and vertical (green) eye position (target in gray), and lower traces indicate horizontal (blue) and vertical (green) eye velocity (°/s). The responses to the step-ramp stimuli (target = red) are shown in **(B)** for a healthy control subject and the patient **(C)**. Note the low initial acceleration in the patient with subsequent catch-up saccades while he pursued the target. **(D)** (from left to right): Mean values (40 ramps) for the latency (ms), the initial acceleration (°/s^2^), and the pursuit maintenance velocity gain are indicated in box plots (with median, upper and lower quartiles, e.g., 75 and 25% percentiles, and outliers) for the healthy subjects (gray) and the patient (green). The distribution of the initial acceleration values (plotting initial acceleration vs. maintenance pursuit velocity) on the right side shows that the low initial acceleration of the patient (green circle) is within the data distribution of the healthy subjects (see also Figure 3).

**Figure 3:**
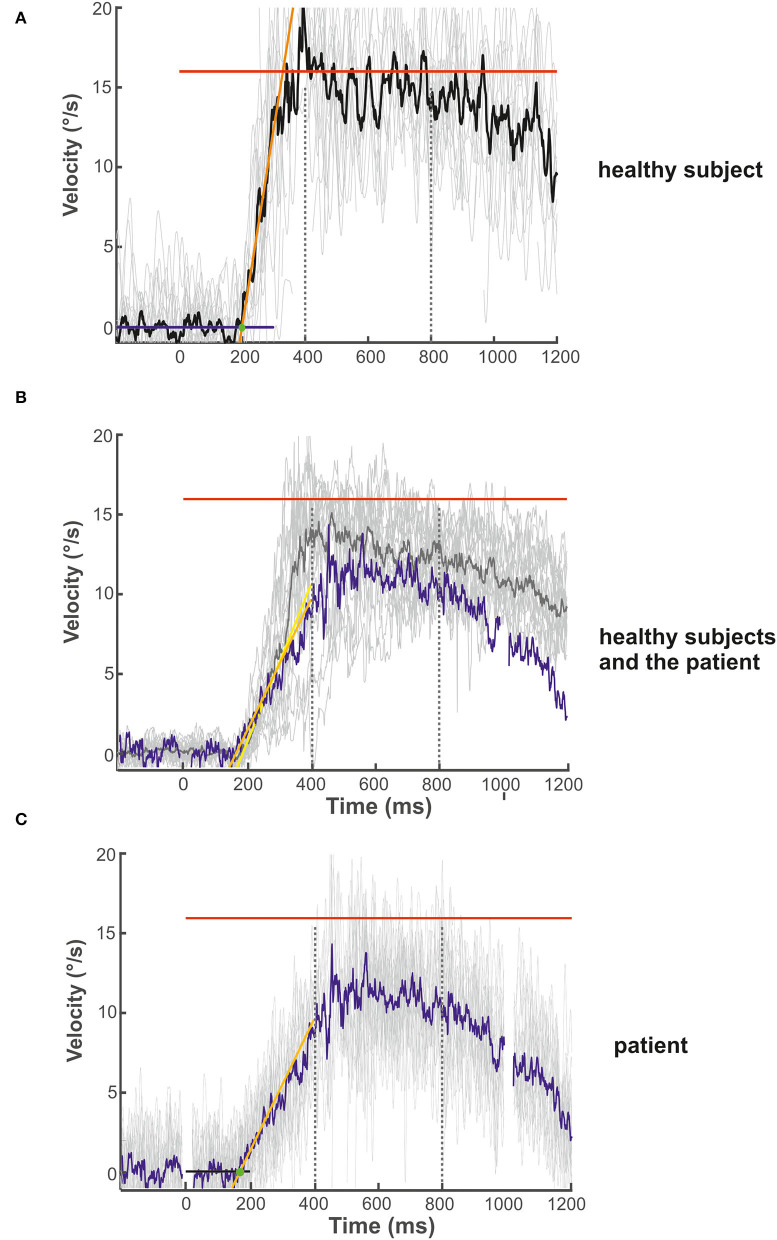
Examples of the initial and maintenance pursuit velocity in a healthy control subject [**(A)**: gain: 0.89, latency: 199 ms, acc.: 123.35 °/s^2^] and the patient [**(C)**: gain: 0.69, latency: 192 ms, acc.: 35.94 °/s^2^] responding to the step-ramp stimulus paradigm. For each subject, the bold trace shows the median of all individual (thin gray lines) ramp velocities. Figure **(B)** shows the mean (thick gray trace) of the individual median eye velocity of all healthy subjects to illustrate the variability of initial acceleration values. For comparison, the blue trace in **(B)** shows the median of the patient's pursuit velocity values, as shown in **(C)**. While there is no difference in pursuit latency, the initial acceleration and velocity gain is low in the patient compared to this healthy control **(C)** but within the data range of the healthy subject group **(B)**. This difference was not significant for the group comparison (see Figure 2D). The target velocity (16°/s) is indicated by the red line. The blue **(A)** and black **(C)** line at the bottom reflects the baseline velocity prior to the ramp, the orange line the regression line of the average pursuit velocity (60 ms after pursuit onset), and the green dot the intersection of the regression line and the baseline before target motion onset. The slope of the orange line indicates pursuit acceleration for both individuals [yellow regression line = mean slope of all healthy subjects in **(B)**]; the time between trial start and the intersection indicates pursuit latency. The interval between both dotted vertical lines was taken for the maintenance pursuit velocity analysis.

Smooth pursuit maintenance was normal in the patient during slow predictive sinusoidal pursuit (0.2 Hz) with a horizontal velocity gain of 0.8 [0.1 Hz: 0.9, 0.3 Hz: 0.68] and 0.7 for the vertical direction [0.1 Hz: 0.96, 0.3 Hz: 0.68], respectively (Figure 2). There was no significant difference to the healthy subjects [*n* = 18, 0.2 Hz: horizontal velocity gain 0.88 ± 0.07; *t*_(17)_ = −1.143, *p* = 0.14, vertical 0.69 ± 0.14; *t*_(17)_ = 0.278, *p* = 0.39]. The patient showed a slightly reduced horizontal steady-state velocity gain (0.69, Figure 3A) during the horizontal step-ramp stimulus, which was not significantly different from the healthy subjects [0.79 ± 0.15; *t*_(17)_ = −0.649, *p* = 0.263]. As pursuit velocity was within normal limits, there were only a few catch-up saccades in the patient, and even less in the healthy subjects. Mean amplitude of catch-up saccades was not different between the patient (3.29 ± 2.08) and the healthy subjects [1.56 ± 1.18; *t*_(17)_ = 1.43, *p* = 0.08].

#### Smooth pursuit initiation in the step-ramp paradigm

The mean latency of horizontal smooth pursuit onset in the foveopetal step-ramp paradigm of the patient (on average 192 ms) was not different from the healthy subjects **[**horizontal: 245 ± 62 ms, *t*_(17)_ = 0.211, *p* = 0.422] (Figure 2D). The initial pursuit acceleration was low (36°/s^2^) but not significantly different from the healthy control subjects [91 ± 43°/s^2^; *t*_(17)_ = 0.117, *p* = 0.234] (Figures 2B–D, 3). Note the variability of initial acceleration in the healthy subjects (Figures 2C,D, 3).

### Saccades

#### Pro-saccades

**Figure 4:**
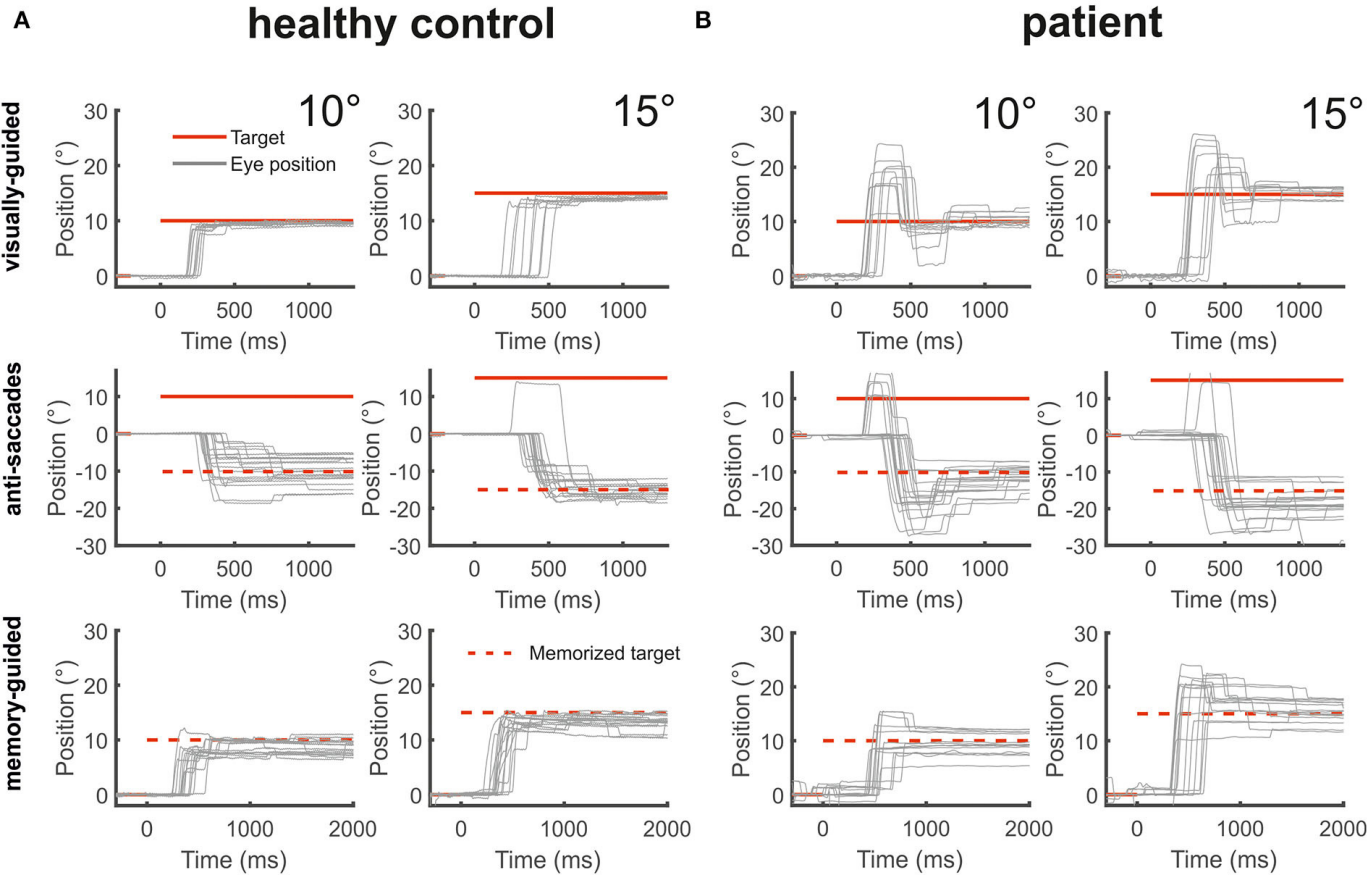
The figure shows from top to bottom horizontal eye position data (gray) during visually-guided saccades (pro-saccades), anti-saccades, and memory-guided saccades for two amplitudes (10 and 15°) for a healthy control [**(A)**, left side] and the patient [**(B)**, right side]. The target at gaze straight ahead or at the defined peripheral location is shown in red lines. The dashed red lines indicate the mirrored target position in the anti-saccade task and the previously presented target position that is to be remembered during the memory-guided saccade task. The patient's saccades during all three saccade tasks were severely hypermetric.

**Figure 5:**
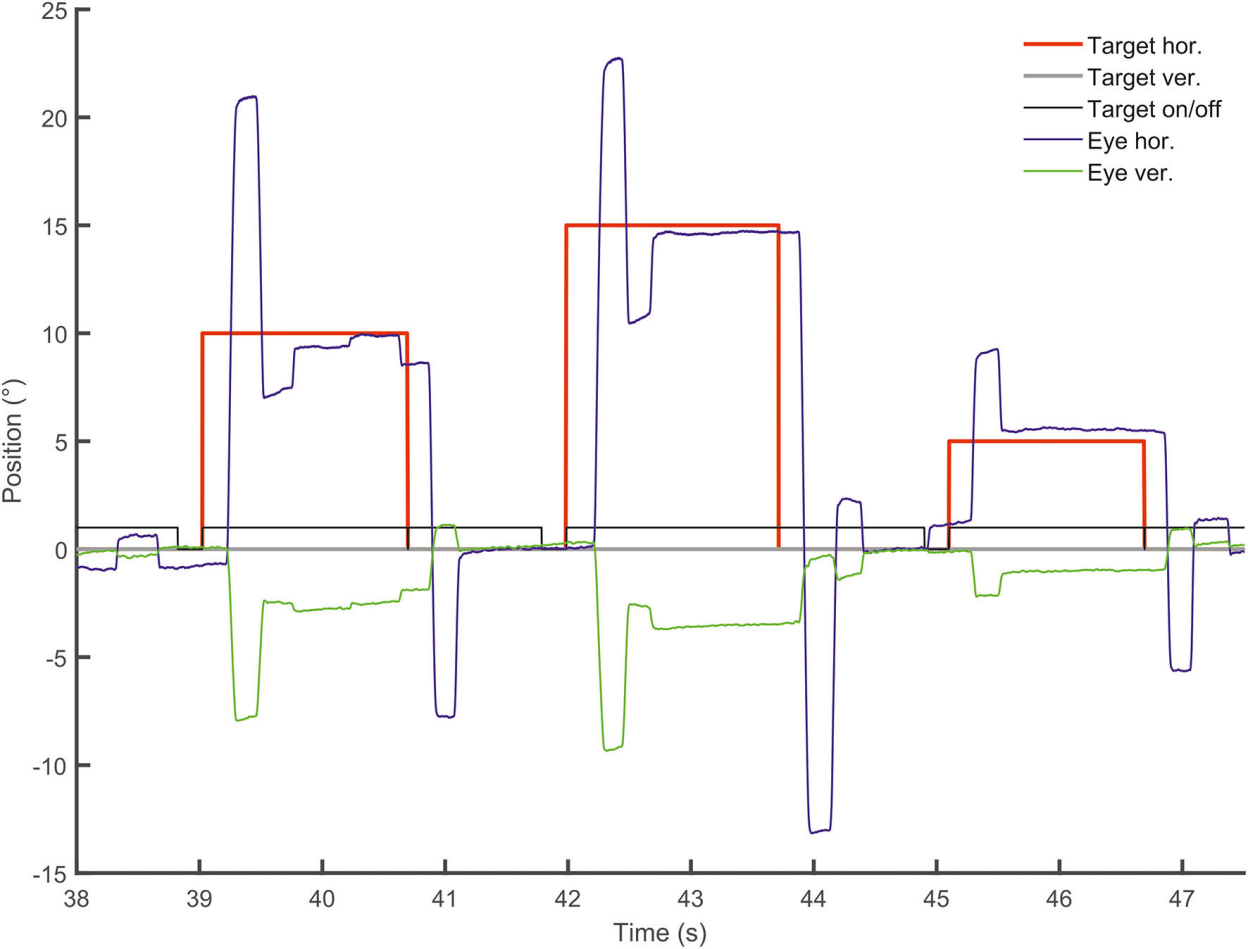
Vertical deflection during horizontal visually-guided pro-saccades. Vertical (green) and horizontal (blue) eye position is shown over time (sec) following horizontal target displacements (red). Note the large vertical deflection during hypermetric horizontal saccades.

Horizontal saccade amplitude gain (30 saccades) was significantly larger (hypermetria) in the patient (gain: 1.59 ± 0.08) than in the healthy subjects [gain 0.96 ± 0.06; *t*_(18)_ = 11.04; *p* < 0.001; Figure 4]. Vertical saccade gain (30 saccades) was also larger (0.98 ± 0.22) compared to the healthy subjects (0.91 ± 0.11) but this difference failed to reach statistical significance (*p* > 0.05). There was often a vertical (usually downward) deflection of the saccade trajectory during horizontal saccades (Figure 5). The majority (93.3%) of all horizontal saccades of the patient were followed by correction saccades (with a median normalized gain of 0.78).

The amplitude of the patient's secondary saccade, i.e., the first correction saccade after the primary saccade, was significantly larger than in the healthy subjects (Table 1, the negative values in the patient reflect a correction in the direction opposite to the hypermetric primary saccade). This larger amplitude of the correction saccade not only results from the different starting point after the primary saccade and its long distance to the target but the correction saccades were considerably larger than required to reach the target, reflecting hypermetria (Figures 4, 5). The gain of the patient's secondary saccade did not differ from the healthy controls (Table 1). The variability of the gain of the first correction saccade was −0.118 ± 0.04.

Horizontal peak velocity of 15° saccades looked normal and did not differ between the patient (398 ± 57°/s) and the healthy subjects (377 ± 48°/s; *p* > 0.05). As the patient made larger (hypermetric) saccades we calculated saccade velocity of a defined amplitude derived from the main sequence, e.g., the patient's peak velocity of a 15° amplitude saccade.

Latency of 30 horizontal pro-saccades of the patient (mean: 214 ± 68 ms) was not different from the healthy control subjects [mean: 182 ± 33 ms; *t*_(18)_ = 0.959; *p* < 0.176].

#### Anti-saccades

**Figure 6:**
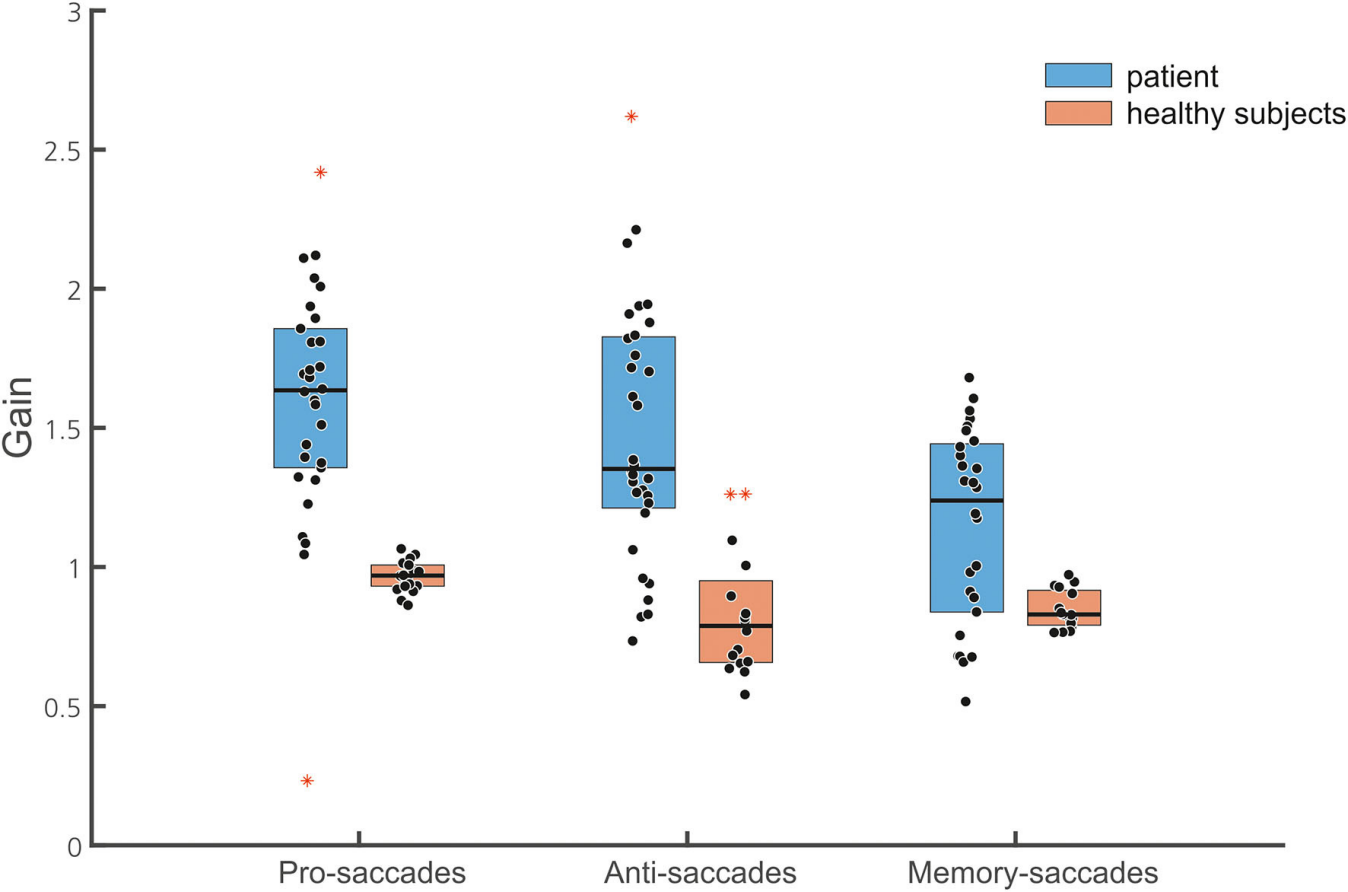
Box plots of the normalized gain of the different saccade tasks are shown (30 pro-saccades, 32 antisaccades, and 28 memory-guided saccades, with marked median; upper and lower quartiles, i.e., 75th and 25th percentiles). Outliers are marked in red. Note that data points of the patient (blue boxes) indicate gain values of single saccades while they represent the mean of the normalized gain of individual subjects in the healthy subject group (brown box). The median gain of all three saccade tasks is larger in the patient than in the healthy subjects.

The patient had an error rate of 20%, i.e., 8 of 40 saccades were directed to the visual target instead of the opposite direction. The patient's rate of misdirected saccades did not differ from the error rate of healthy subjects [24%; *t*_(16)_ = 0.321; *p* > 0.05]. The amplitude gain of horizontal anti-saccades (*n* = 32) was significantly larger in the patient (1.47 ± 0.45) than in the healthy subjects (*n* = 30) [0.83 ± 0.223; *t*_(16)_ = −2.815, *p* = 0.007; Figures 4, 6]. Almost half (45%) of all horizontal anti-saccades of the patient were followed by correction saccades. The number of anti-saccades with correction saccades was not different from the healthy subjects [54 ± 2%, *t*_(16)_ = −0.42; *p* > 0.05]. The gain of the patient's first correction saccade did not differ from the healthy controls (Table 1). The mean amplitude of the patient's first correction saccade was also significantly larger than in the healthy subjects (Table 1), with correction saccades in the direction opposite to the primary saccade.

Latency of anti-saccades of the patient (mean: 400 ± 72 ms) was not different from the healthy control subjects [mean: 328 ± 53 ms; *t*_(16)_ = 1.29; *p* = 0.109].

#### Memory-guided saccades

The frequency of reflexive saccades (toward the memorized target at 10 or 15°) was not different between the patient (16.6%) and the healthy subjects [16% ± 11.5; *t*_(15)_ = 0.049, *p* = 0.481]. The gain of the patient's primary memory-guided saccades was significantly larger than in the healthy participants (Figure 6). The gain of the patient's first correction saccade toward the memorized target was not different from the healthy subjects (Table 1). The proportion of correction saccades was not different between the patient (in 16/30 of saccades; i.e., 53% of all saccades) and the healthy subjects [66 ± 17.7%, *t*_(16)_ = 0.726; *p* = 0.24]. Apart from square wave jerks during fixation (0.5–2°, 200 ms duration; only found in the patient's records), there were neither macrosaccadic oscillations nor irrepressible saccades.

The final eye position gain did not differ between the patient (0.98 ± 0.26) and the healthy subjects [0.93 ± 0.05, *t*_(16)_ = 1.008; *p* = 0.165]. The variability of the final eye position (standard deviation of the gain of each single subject) was not different between the patient (0.26) and the healthy subjects [0.19 ± 0.11; *t*_(16)_ = 0.626, *p* = 0.27]. The latency of the first saccade of the patient (452 ± 110 ms) was not different from the healthy participants [322 ± 100 ms, *t*_(15)_ = 1.257, *p* = 0.115].

Unfortunately, a statistical within-subject comparison for pro-saccades with memory-guided saccades is technically limited due to the case nature of this study. As a 2 × 3, ANOVA [two groups, three saccade tasks (pro/anti/memory-guided saccades)] is technically not valid, we can only state trends. They are based on an ANOVA analysis of each group (patients and healthy subjects) separately comparing the three different saccade conditions (Figure 6). For the patient, the ANOVA of the primary saccade gain showed a significant main effect for the saccade tasks [*F*_(2, 87)_ = 8.955, *p* < 0.001]: saccade amplitude of memory-guided saccades was smaller compared to anti-saccades (*p* = 0.008) and pro-saccades (*p* < 0.001).

#### Fixation and gaze holding

During fixation, there were numerous square wave jerks. We neither found irrepressible saccades nor macrosaccadic oscillations. There was no spontaneous nystagmus and or gaze-evoked nystagmus during sustained eccentric vertical and horizontal fixation for at least 20 s in the light and darkness. After eccentric fixation (20 s), there was no rebound nystagmus.

## Discussion

Our main goal was to investigate the effects of bilateral lesions of the deep cerebellar nuclei on the initial smooth pursuit acceleration.

As a main result, initial pursuit acceleration was low but not statistically different from the healthy control subjects, as was the predictive (sinusoidal) pursuit velocity. Bilateral saccade hypermetria was not only seen in visually-guided saccades but also in anti-saccades and memory-guided saccades. The final eye position remained accurate. We will first describe the lesioned cerebellar structures of our patient, relate them to mechanisms causing the pursuit and saccade abnormalities, and finally elaborate on the dysmetria of his volitional saccades, i.e., antisaccades and memory-guided saccades.

The patient's hemorrhage was centered in the midline between the deep cerebellar nuclei and extended rostrally into the vermal lobules IV and V and the right hemispheric lobules V and VI (40, 41). The edema bilaterally involved the fastigial nuclei anteriorly to the hemorrhage at the roof of the fourth ventricle, most likely impairing inter-fastigial projections (15), and laterally the interpositus nuclei (globose and emboliform nuclei) and the medial part of the dentate nuclei (Figure 1). The lesion did not comprise hemispheric lobule VI (simple lobule), the posterior oculomotor vermal lobules VI and VI (OMV), flocculus, paraflocculus, and caudal vermal uvula and nodulus. Functionally, the only lesion site in the whole brain known to elicit such a striking bilateral saccade hypermetria is a bilateral impairment of the FOR.

### Smooth pursuit eye movements

Experimental unilateral FOR lesions in non-human primates elicit severe contralesional smooth pursuit impairment comparable in magnitude to the deficit observed during floccular lesions (48). Unilateral FOR lesions in the non-human primates not only impair contralateral but also increase ipsilesional pursuit acceleration (4) as the FOR neurons burst throughout the initial third of the eye acceleration with a subsequent steady firing (13). Moreover, it has been proposed that unilateral FOR lesions change the bilateral balance of pursuit-related activity in the recipients from the FOR (49), either directly in the pontine or indirectly in the ventral posterolateral thalamic nuclei (50, 51). Accordingly, the bilateral equilibrium is impaired by the asymmetrical FOR input (1, 49). According to this equilibrium hypothesis, the contralesional (e.g., right) pursuit impairment after unilateral (i.e., left-sided) FOR lesions is functionally related to the unilateral suppression of the pursuit-related activity in the pontine and thalamic nuclei (1).

In bilateral FOR lesions, however, the impaired contralesional pursuit acceleration is counterbalanced by the impaired ipsilesional pursuit deceleration making the pursuit maintenance phase appear normal (4). Thus, both fastigial nuclei would dynamically adjust the balance (symmetry) of directional smooth pursuit premotor commands (1, 49, 52). In bilateral lesions, the pontine and thalamic pursuit-related structures do not receive any or very little symmetrical action potentials from the FOR.

Bilateral FOR lesions in non-human primates (4) showed normal pursuit latency and sinusoidal pursuit gain was normal toward the ipsilesional direction and only slightly reduced toward the contralesional direction. In unilateral FOR lesions, pursuit acceleration was found to be increased to the ipsilesional side or decreased to the contralesional side. Subsequent inactivation of the contralateral FOR, functionally resulting in bilateral FOR lesions, normalized pursuit acceleration as FOR activity that aids contralateral and reduces ipsilateral acceleration, was abolished. Accordingly, bilateral FOR lesions restore the imbalance of opposing driving pursuit forces that are impressively seen in unilateral FOR lesions. Thus, a deficient pursuit acceleration due to a unilateral FOR lesion can be normalized by an additional contralateral FOR lesion (bilateral FOR lesion).

It has therefore been proposed that the pursuit acceleration is generated outside the FOR (4). A study on a cohort of stroke patients with unilateral cerebellar lesions revealed impaired pursuit acceleration to the ipsilesional side but only some (5 of 10) patients had lesions spreading into the FOR (14). Noticeably, pursuit acceleration to the ipsilateral side was impaired while experimental unilateral FOR inactivations impaired contralateral pursuit acceleration (4). In contrast, bilateral FOR inactivation in non-human primates did not impair the initial acceleration of smooth pursuit, i.e., the open-loop period of smooth tracking behavior (first 100 ms) (4). In line with these animal data, the initial acceleration of smooth pursuit of our patient was at the lower level of the normal reference range in related studies, e.g., 42 ± 6°/s^2^ (53), 40–100°/s^2^ (54) or 44–124°/s^2^ (55). The difference with our control group did not reach statistical significance. Therefore, this result should be confirmed in a larger cohort of patients with confined FOR lesions. As they are very rare, we felt compelled to report this first recording of the initial pursuit acceleration in this patient.

Instead, reduced initial acceleration is found in posterior vermal lesions in non-human primates (7) with moderately impaired sustained pursuit (56) suggesting that the oculomotor vermis plays a critical role in the online control of pursuit (7, 57).

Clinically, vermal lesions cause a decrease of ipsilateral smooth pursuit gain (56), which was not found in our patient. The lesion of our patient affected the FOR bilaterally but clearly did not involve the OMV (vermal lobules VI, VII). We cannot, however, rule out that the hemorrhage or its edema damaged the inhibitory control of the Purkinje cell fibers from the OMV (lobules VIIA and VIc) to the smooth pursuit neurons in the FOR. This functional impairment could have contributed to the low initial pursuit acceleration. Other cerebellar structures controlling smooth pursuit were not lesioned, even by edema, in particular the flocculus and paraflocculus (48, 58, 59).

Lesions of bilateral interpositus (globose and emboliform) nuclei, which were involved in our patient's edema, slightly affect vertical smooth pursuit and vertical saccades but not horizontal pursuit and saccades (60). The hemispheric oculomotor region (HOR) is a relatively large region that extends from the OMV into the simple lobules of the cerebellar hemispheres (12) which were spared in our patient's lesion. Up to now, there is no evidence that lesions of the dentate or vermal lobules IV and V (being affected by the patient's hemorrhage) control visually-guided goal-directed eye movements.

### Visually-guided pro-saccades

Dysmetria of visually-guided saccades (VGS) in cerebellar disease is caused by midline lesions affecting the OMV or the FOR. While vermal lesions elicit hypometric saccades (6, 21), FOR lesion causes contralesional saccade hypometria and ipsilesional hypermetria when it is unilateral, bilateral hypermetria when it is bilateral (1, 2, 5, 22). In line with previous clinical reports (11, 34) our patient showed severe bilateral saccade hypermetria when visual information about the target was provided. Evidence for some visual influence on saccade dysmetria comes from the following observations in patients: (i) saccade dysmetria is heavily dependent on visual signals as it may disappear in darkness (61); (ii) correction saccades are dysmetric (62), in line with our patient, i.e., his consecutive correction saccades (in contrast to the non-visually guided saccades) remained hypermetric until he reached the visible target; and (iii) dysmetria increases once the target jumps from one to another target in brief succession (double-step paradigm) (63) suggesting impaired neural parallel processing of saccadic signals in cerebellar disease (64). Noticeably, saccades of non-human primates toward flashed peripheral visual targets in complete darkness were still dysmetric (22, 23).

It has been proposed that bilateral saccade hypermetria results from a faulty feedback control of saccades in the brain stem, manifesting with an impairment to accelerate contralateral and decelerate ipsilateral saccades (5). Accordingly, bilateral hypermetria in bilateral FOR lesions results from both impaired saccade acceleration and deceleration. In line with experimental FOR lesions hypermetria of vertical saccades and vertical deflection during horizontal saccades were smaller in our patient (Figure 5). The latency and saccade velocity of our patient's visually-guided saccades were normal, in accord with experimental FOR lesions (5). The patient‘s final eye position was on target, i.e., there was no increased end-point variability as seen in cerebellar disease which has been attributed to an increase of accumulating signal noise (65) or a feedforward rather than feedback saccade control, respectively (66, 67).

The mechanisms for the saccade and pursuit disorders in experimental FOR lesions seem to be unrelated as they were not correlated to each other (1) suggesting that caudal FOR processes saccade and pursuit signals independently.

This visual influence implies that saccade hypermetria may decrease or disappear in the absence of a visual target. But even visual objects may not necessarily elicit saccade dysmetria as it is not found during visual scanning (8). Therefore, we examined memory-guided saccades and antisaccades (29). Although initiated by a visual cue, the neural drive of these volitional saccades is based on a calculated but not a visible target position. Subjects had to look at an imagined target position during the anti-saccade paradigm without having seen a visual target at this location. In the memory-guided paradigm, they had to keep the visible target position in mind within a variable interval challenging working memory. Thus, the execution follows a neural representation of a previously shown visible target that is no longer visible anymore.

### Memory-guided saccade and anti-saccades

Up to now, three studies investigated memory-guided saccades in cerebellar disease but only a few patients had lesions involving the FOR (10, 26, 34). As memory-guided saccades (MGS) were equally hypermetric compared to VGS, the authors proposed that these cerebellar patients were unable to use feedforward internal signals (e.g., efference copy signals) to estimate final eye position (34). In our patient, there was a trend toward a lesser hypermetria in MGS and anti-saccades compared to VGS. Correction saccades following the first MGS were hypometric while those directed toward the reilluminated target in the gaze straight ahead position were always hypermetric. Accordingly, the gain of the correction saccades was also smaller in MGS and anti-saccades compared to VGS. Noticeably, the proportion of correction saccades in the MGS task did not differ from healthy subjects but its direction differed: while correction saccades of the patient were directed backward toward the memorized target (due to hypermetria), it was directed toward it in the healthy participants due to physiological hypometria (62). However, the final eye position after correction saccades was normal indicating preserved internal spatial representation of the target. This is in contrast to FOR patients who maintained their gaze toward the erroneous eye position until the target was switched on again (34). Our patient seems to be able to use internal (e.g., efferent copy) signals to control saccade accuracy which is in line with previous data (63). Both cerebellar midline structures (the FOR and the OMV) contain not only saccade-related neurons that are more active in the light compared to darkness (19, 27) but also a type of neurons whose discharge is unrelated to eye movements but to the memory of a previously seen smooth pursuit target as they discharge in the “no-go instruction” period (33). The authors suggested that this pathway contributes specifically to motor planning engaging the working memory of no-go instructions and the preparation of tracking eye movements. It is supposed to be part of the cerebro-cerebellar loops (involving the supplementary eye field and the pontine nuclei) for no-go instruction working memories and is engaged in the decision of whether or not to, and what to pursue. Unfortunately, it is unknown how these cells respond to MGS. Recently, new projections from the deep cerebellar nuclei to the hippocampus *via* the thalamic nuclei have been identified in mice that may subserve memory-related cognitive functions (68). In the posterolateral region of the thalamus, pursuit-related neurons discharge before and during the initiation of ipsiversive pursuit and may receive projections from the contralateral FOR (1, 50, 51).

Anti-saccades have not been studied in FOR lesions before. Similar to MGS, our patient's anti-saccades were also hypermetric, with a trend to larger amplitudes compared to MGS and smaller compared to VGS. The execution of anti-saccades not only requires suppression of reflexive involuntary saccades to the target but to mirror and memorize the visual target position which clearly engages working memory as well. Correction saccades were hypometric compared to those of hypermetric VGS and larger compared to those of healthy control subjects. As the final eye position did not differ from controls, the working memory of the target location and of its mirrored position was intact. Along with the normal latency and error rate of anti-saccades disease-related cognitive (e.g., attention) deficits are unlikely to contribute to saccade dysmetria.

In conclusion, we provide some clinical evidence that a bilateral lesion of the deep cerebellar nuclei does not impair the initial acceleration of smooth pursuit, as it is found in unilateral FOR lesions. This result is in line with experimental FOR lesions in non-human primates. At the same time, the lesion caused severe saccade hypermetria. The neural correlate of peripheral target locations seems to remain unimpaired in our patient and in fact, contributes to the pronounced hypermetria of MGS and antisaccades. In light of the dissociation of moderately preserved initial and maintenance smooth pursuit but severe saccade hypermetria, our data argue against an impaired common command feeding the circuits controlling saccadic and pursuit eye movements. Instead, they are consistent with independent influences on the neural processes generating both eye movements (1).

## Data availability statement

The raw data supporting the conclusions of this article will be made available by the authors on request.

## Ethics statement

The studies involving human participants were reviewed and approved by the Ethics Committee of the University of Lübeck. Written informed consent for participation was not required for this study in accordance with the national legislation and the institutional requirements.

## Author contributions

CH: design or conceptualization of the study, analysis and interpretation of the data, and drafting the manuscript. BM: interpretation of the data and revising the manuscript for intellectual content. HS: analysis or interpretation of the data and revising the manuscript for intellectual content. AS: acquisition, analysis, and interpretation of the data, and revising the manuscript for intellectual content. All authors contributed to the article and approved the submitted version.

## Conflict of interest

The authors declare that the research was conducted in the absence of any commercial or financial relationships that could be construed as a potential conflict of interest.

## Publisher's note

All claims expressed in this article are solely those of the authors and do not necessarily represent those of their affiliated organizations, or those of the publisher, the editors and the reviewers. Any product that may be evaluated in this article, or claim that may be made by its manufacturer, is not guaranteed or endorsed by the publisher.
